# Mesenchymal stromal cell-derived extracellular vesicles (MSC-EVs) as a novel topical immunomodulatory therapy for psoriasis: bridging the therapeutic gap in moderate disease

**DOI:** 10.20517/evcna.2025.150

**Published:** 2026-04-24

**Authors:** Thong Teck Tan, Kok Hian Tan, Sai Kiang Lim

**Affiliations:** ^1^Paracrine Therapeutics Pte. Ltd., Singapore 536464, Singapore.; ^2^Division of Obstetrics and Gynaecology, KK Women’s and Children’s Hospital, Singapore 229899, Singapore.; ^3^Department of Surgery, Yong Loo Lin School of Medicine, National University of Singapore, Singapore 119228, Singapore.

**Keywords:** Psoriasis, extracellular vesicles, mesenchymal stromal cells, immunomodulation, complement system, IL-17, topical therapy, cell-free therapeutics

## Abstract

Psoriasis is a chronic, immune-mediated inflammatory disease that affects approximately 2%-3% of the global population, and remains a major dermatologic and psychosocial burden. Despite advances in biologics targeting interleukin 17 (IL-17) and interleukin 23 (IL-23) pathways, effective and accessible treatment options for moderate psoriasis are lacking. Topical therapies and phototherapy are often inadequate, while systemic agents and biologics are limited by toxicity, high cost, and restricted reimbursement criteria, leaving patients with moderate disease without adequate therapeutic options. Mesenchymal stromal cell-derived extracellular vesicles (MSC-EVs) have emerged as a promising acellular therapeutic modality that harnesses the immunomodulatory and regenerative properties of parent MSCs. Unlike systemic biologics, MSC-EVs act locally and non-immunosuppressively. Topically applied MSC-EVs have demonstrated the ability to modulate cutaneous inflammation by attenuating complement activation [via CD59-mediated inhibition of complement terminal component 5b-9 (C5b-9) complex formation], reducing neutrophil infiltration, and subsequently lowering IL-17 and IL-23 expression in psoriatic lesions. Preclinical and early clinical studies suggest that MSC-EVs can restore local immune homeostasis through paracrine extracellular mechanisms, without systemic absorption or adverse effects. MSC-EVs represent a new class of cell-free nanotherapeutics inspired by biologics, capable of localized immunomodulation in psoriasis. By combining biologic-like efficacy with the safety and accessibility of topical therapy, MSC-EVs may bridge the long-standing therapeutic gap in moderate psoriasis. This review discusses current treatment limitations, the mechanistic rationale for MSC-EVs in psoriatic inflammation, and their potential to redefine dermatologic immunotherapy.

## INTRODUCTION

Psoriasis is a chronic, immune-mediated inflammatory skin disorder affecting approximately 2%-3% of the global population, or more than 125 million individuals worldwide. Prevalence varies among racial and ethnic groups, with the highest prevalence in Caucasians (~3.6%) and lowest in African Americans (~1.5%). The disease typically exhibits a bimodal distribution of onset, with incidence peaks in early adulthood (20-30 years) and later middle age (50-60 years).

Beyond being a skin disorder with its characteristic erythematous, scaly plaques, psoriasis is a systemic inflammatory disease linked to complications such as metabolic syndrome, hypertension, type 2 diabetes, cardiometabolic disease, psychological illnesses, and inflammatory bowel diseases (https://www.psoriasis.org/about-psoriasis/)^[1,2]^. *Psoriasis vulgaris*, the most prevalent form, affects approximately 90% of psoriatic patients^[3,4]^. Between 1990 and 2021, the global Disability-Adjusted Life Years (DALYs) attributable to psoriasis increased from approximately 2.0 million to 3.69 million - an 85% rise^[5]^. According to the World Health Organization (WHO), psoriasis is recognized as a serious, chronic, noncommunicable disease that causes painful, itchy, and disfiguring skin lesions and is frequently associated with multiple comorbidities, including metabolic syndrome, cardiovascular disease, and depression.

Access to effective care remains inequitable, limited by high treatment costs, insurance restrictions, and insufficient dermatologic service^[6]^. These barriers disproportionately affect underserved populations, exacerbating disease burden and health disparities. Given its chronicity, systemic immune involvement, and lifelong impact, there is an urgent need for safe, effective, and accessible therapies that not only control disease activity but also improve quality of life and promote health equity.

### Current treatment landscape

Psoriasis typically presents as erythematous, scaly plaques on the elbows, knees, scalp, and trunk^[7]^. Therapeutic selection is guided by severity, lesion distribution, comorbidities, and patient preference, with stratification frameworks provided by regulatory and clinical bodies such as the National Institute for Health and Clinical Excellence (NICE), the National Psoriasis Foundation (NPF) and American Academy of Dermatology (AAD)^[8,9]^.

#### Treatment stratification in psoriasis

Alignment with NICE (Guidelines 2025) and AAD (Guidelines 2025). Both NICE (UK) and AAD (US) recommend a stepwise approach to psoriasis therapy based on disease severity, lesion distribution, comorbidities, and patient preference.

Mild disease - topical therapies First-line for localized psoriasis. Corticosteroids, vitamin D analogues, calcineurin inhibitors, retinoids, and keratolytics remain standard. Combination corticosteroid-calcipotriol improves efficacy and reduces steroid burden. Newer non-steroidal agents - tapinarof (AhR agonist) and roflumilast [Phosphodiesterase type 4 (PDE4) inhibitor] - provide steroid-sparing alternatives, especially for sensitive sites^[10,11]^. Limitations include adherence challenges, skin atrophy with chronic steroids, and cost/access barriers for newer agents.

Moderate disease - phototherapy Narrowband Ultraviolet B (UVB) is the preferred modality, achieving Psoriasis Area and Severity Index (PASI) 75 in ~60%-70% within 12-16 weeks^[12]^. PUVA (Psoralen + Ultraviolet A) is effective but carries a carcinogenic risk, while excimer lasers are useful for localized or refractory cases; both are limited by accessibility. Phototherapy is often impractical as it requires multiple weekly clinic visits, and relapse is common after discontinuation.

Moderate disease - systemic non-biologics Recommended when topicals and phototherapy fail. Methotrexate (PASI 75 in ~40%-50%)^[13,14]^ and cyclosporine (rapid control but short-term) are widely used but constrained by hepatotoxicity, nephrotoxicity, teratogenicity, and monitoring requirements. Newer oral small molecules - apremilast (PDE4 inhibitor) and deucravacitinib [Tyrosine kinase 2 (TYK2) inhibitor] - offer improved safety and convenience but only modest efficacy (PASI 75 ~30%-50%)^[15,16]^.

Moderate-to-severe disease - biologics Reserved for severe psoriasis [e.g., PASI ≥ 10, Dermatology Life Quality Index (DLQI) > 10] under both NICE and AAD criteria. Interleukin 23 (IL-23) (guselkumab, risankizumab) and IL-17 (secukinumab, ixekizumab) inhibitors provide the most robust efficacy, with PASI 90 achieved in > 70%-80% of patients^[17]^. However, systemic immunosuppression increases infection risk, paradoxical inflammation may occur, and immunogenicity can lead to loss of response. High costs and strict reimbursement criteria further restrict access, particularly for moderate disease.

**Figure 1:**
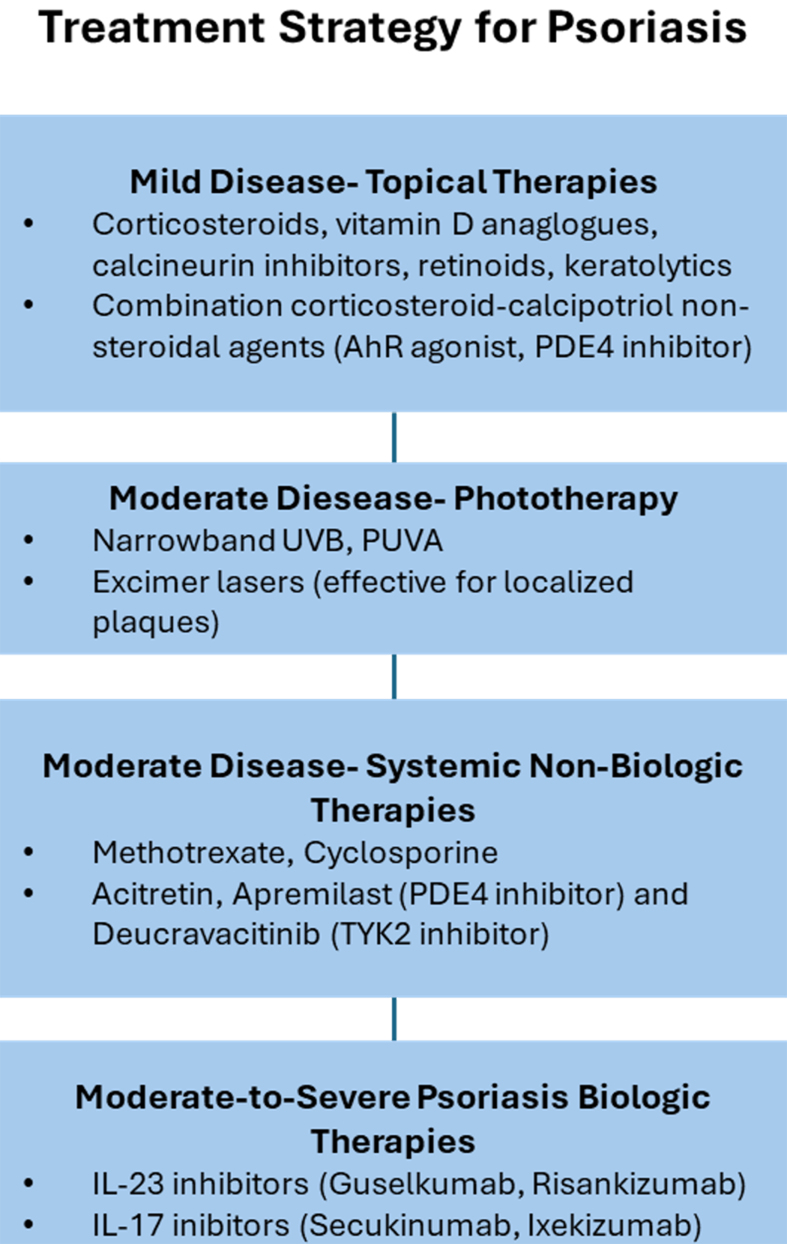
Treatment Strategy for Psoriasis: Stepwise Approach Based on Disease Severity. This schematic summarizes the current therapeutic hierarchy for psoriasis, illustrating treatment escalation from mild to severe disease. Mild psoriasis is managed primarily with topical therapies such as corticosteroids, vitamin D analogues, calcineurin inhibitors, retinoids, keratolytics, and newer non-steroidal agents (AhR agonists, PDE4 inhibitors). For moderate disease, phototherapy (narrowband UVB, PUVA, excimer laser) and systemic non-biologic agents (methotrexate, cyclosporine, acitretin, apremilast, deucravacitinib) are employed. In moderate-to-severe cases, biologic therapies targeting IL-23 (guselkumab, risankizumab) and IL-17 (secukinumab, ixekizumab) provide the highest efficacy and durability. AhR: Aryl hydrocarbon receptor; PDE4: phosphodiesterase 4; UVB: ultraviolet B; PUVA: psoralen plus ultraviolet A; TYK2: tyrosine kinase 2; IL-23: interleukin-23; IL-17: interleukin-17.

Current treatment stratification, aligned with NICE and AAD guidelines, ensures that mild and severe psoriasis are well addressed [Figure 1]. However, patients with moderate psoriasis remain underserved, caught between inadequate topicals/phototherapy and costly biologics restricted to severe disease. This therapeutic gap highlights the need for novel, safe, and accessible topical interventions with minimal systemic toxicity.

## NOVEL THERAPEUTIC OPTION FOR UNDERSERVED MODERATE PSORIASIS

**Figure 2:**
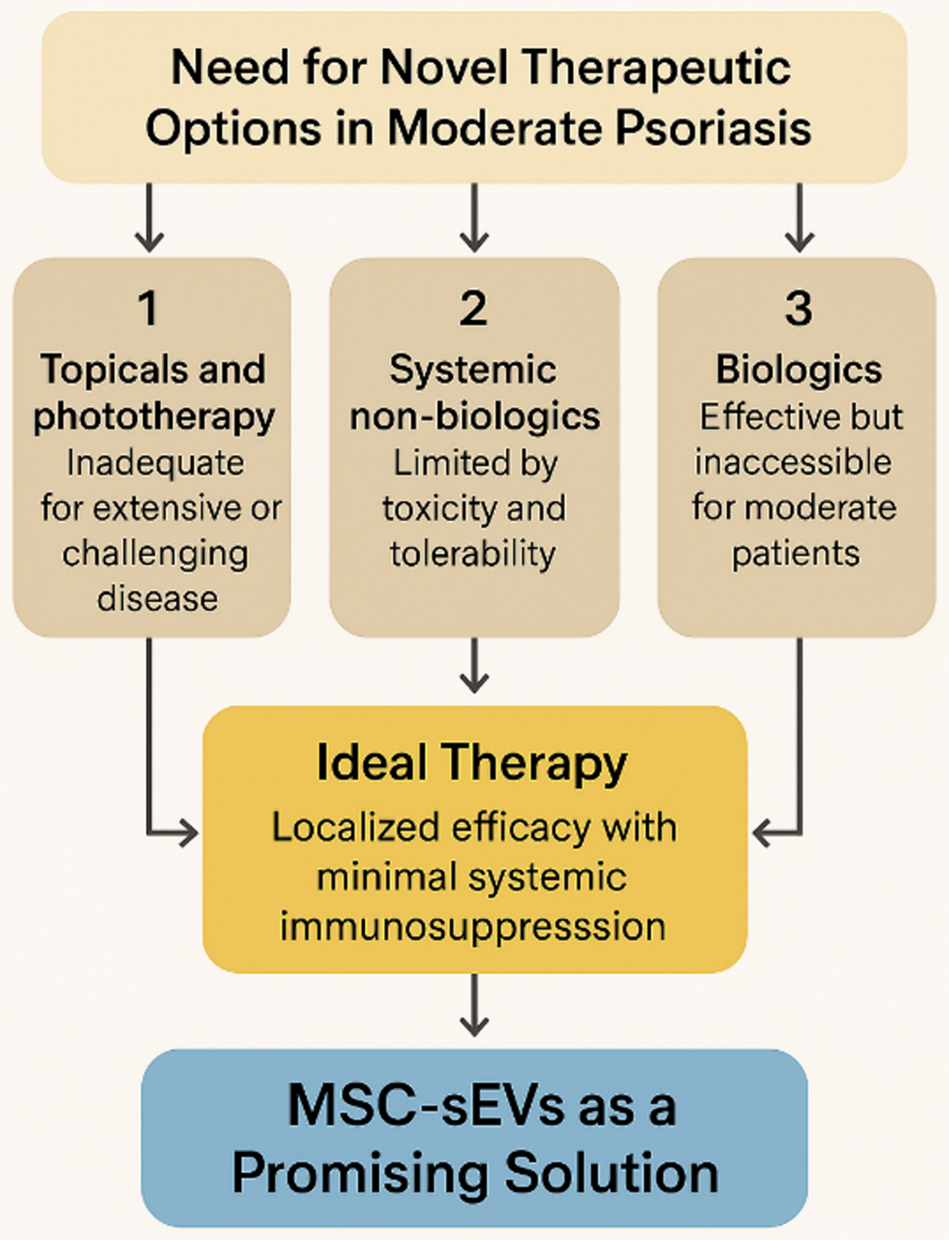
Addressing the Therapeutic Gap in Moderate Psoriasis with MSC-sEVs. Schematic summary of current therapeutic limitations in moderate psoriasis. Topical agents and phototherapy are inadequate for widespread or high-impact disease, while systemic non-biologics are constrained by toxicity and monitoring burdens. Biologics remain highly effective but are inaccessible for most moderate patients due to cost, systemic risks, and payer restrictions. MSC-sEVs represent a novel, cell-free, topical therapy capable of localized immunomodulation with minimal systemic toxicity, offering a scalable and patient-friendly solution to this critical therapeutic gap. (Figure was created with AI-assisted technologies). MSC-sEVs: Mesenchymal stem cell-derived small extracellular vesicles.

Psoriasis management is typically stratified by disease severity: topicals and phototherapy for mild cases, systemic non-biologics for moderate disease, and biologics for severe presentations. While this framework is well established, patients with moderate psoriasis remain inadequately treated. Topical therapies and phototherapy are often insufficient for extensive or high-impact lesions and are constrained by adherence, access, and cumulative risk. Conventional systemic agents (e.g. methotrexate, cyclosporine) can induce short-term control but are limited by hepatotoxicity, nephrotoxicity, teratogenicity, and monitoring requirements, rendering them suboptimal for long-term use^[18]^. Biologic therapies (e.g. IL-17 and IL-23 inhibitors) deliver durable, high-level clearance but are expensive, require parenteral administration, and are typically reimbursed only for severe disease (e.g. PASI ≥ 10, DLQI > 10) in many health systems. This therapeutic gap underscores the need for novel, safe, and accessible topical interventions with minimal systemic toxicity for patients with moderate psoriasis [Figure 2].

An ideal therapy for moderate psoriasis would be one that combines localized efficacy with minimal systemic immunosuppression to achieve durable disease control without the risks, cost, or access barriers associated with biologics. A topical biologic-inspired therapy that provides targeted immunomodulation while maintaining safety and scalability would directly address this unmet need - particularly for patients’ ineligible for systemic biologics due to medical or economic constraints.

## MESENCHYMAL STROMAL CELL-DERIVED EXTRACELLULAR VESICLES AS A PROMISING SOLUTION

**Figure 3:**
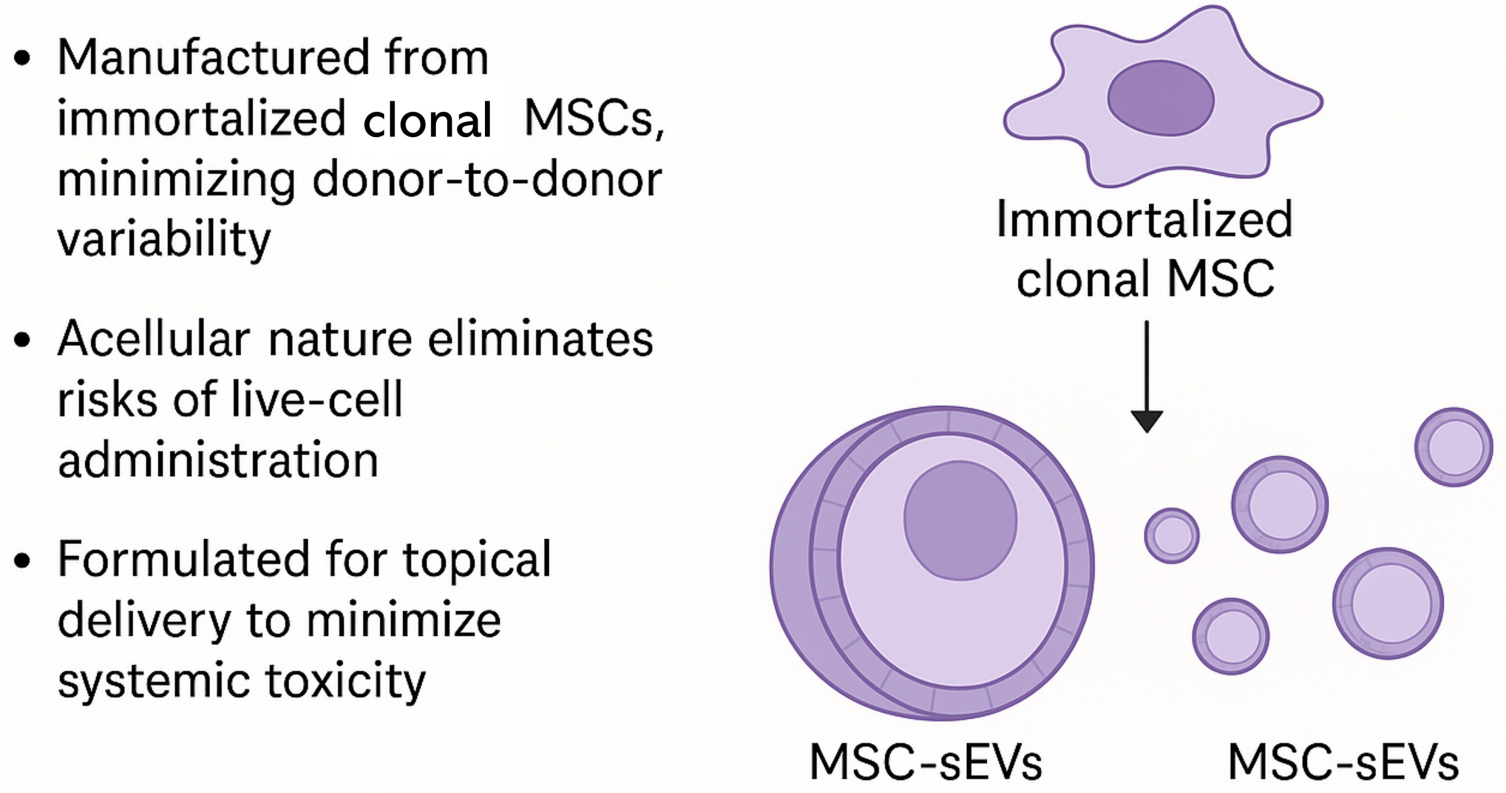
A Cell-Free and Scalable Therapeutic Modality: MSC-sEVs as a New Class of Biologic-Inspired Therapeutics. This graphical abstract illustrates the unique advantages of MSC-sEVs as cell-free, biologic-inspired therapeutics applied topically to psoriatic skin. MSC-sEVs offer localized immunomodulation with minimal systemic exposure, effectively bridging the therapeutic gap in moderate psoriasis. Unlike conventional biologics or live-cell therapies, MSC-sEVs can be manufactured at scale from immortalized monoclonal MSC lines, ensuring product consistency and overcoming donor variability. This platform enables biologic-like efficacy with enhanced safety, accessibility, and scalability. MSCs: Mesenchymal stem cells; sEVs: small extracellular vesicles.

Mesenchymal stromal cell-derived extracellular vesicles (MSC-EVs) are emerging as a novel therapeutic modality uniquely suited to address the unmet need in moderate psoriasis [Figure 3].

### Biological basis and safety

MSCs are among the most clinically studied cell types, with over 1,800 registered trials (as of June 2025) exploring their immunomodulatory properties. These studies have consistently demonstrated their safety. Meta-analyses of MSC therapy have shown that MSC administration does not increase the rate of serious adverse events (SAEs) compared with controls, with most reported adverse events (AEs) being mild and transient (e.g., low-grade fever, infusion-related reactions)^[19]^. Importantly, subsequent mechanistic work has revealed that much of their therapeutic activity is mediated through secreted vesicles rather than direct cell engraftment^[20-22]^. MSC-sEVs are nanosized (50-200 nm) lipid bilayer vesicles secreted by MSCs as part of their paracrine signaling repertoire. MSC-sEVs recapitulate many of the parent cells’ immunomodulatory and anti-inflammatory effects^[23,24]^, supporting their potential utility in psoriasis - an immune-mediated inflammatory disease. A 2025 meta-analysis of 48 studies (1,773 patients, including 6 EV trials) found no increased risk of AEs or SAEs with EV interventions compared to standard care^[25]^. In contrast, conventional systemic therapies used for moderate psoriasis have well-characterized toxicity and AE profiles as stated in section “Current Treatment Landscape”. MSC-sEVs are non-replicative, minimally immunogenic, and - when applied topically - remain confined to the stratum corneum without detectable systemic absorption (Sections “Biodistribution of Topical MSC-sEVs” and “Phase 1 Clinical Evidence”). These characteristics support the proposition that topical MSC-sEVs may offer a more favourable safety profile than conventional systemic therapies, although this requires confirmation in future comparative clinical trials.

### Advantages over MSC cell therapies

While MSC-based cell therapies and MSC-sEVs share overlapping immunomodulatory mechanisms, several evidence-based considerations support the use of MSC-sEVs as a more suitable modality for topical treatment of psoriasis.

(1) Conventional MSC products are typically derived from primary donor cells with a finite lifespan and substantial donor-to-donor and tissue-to-tissue variability in phenotype and secretory profile. In contrast, MSC-sEVs can be produced from immortalized monoclonal MSC lines, which enables consistent, large-scale manufacturing of vesicles from a genetically and phenotypically stable cell source, ensuring reproducibility and product uniformity^[26-28]^.

(2) MSC infusions are generally safe but retain theoretical and observed risks intrinsic to live-cell products, including vascular occlusion, long-term tumorigenic potential and immune rejection. MSC-sEVs, by contrast, are non-replicative, with minimal expression of MHC class I/II, and thus have a lower intrinsic risk of allo-immunogenicity or malignant transformation^[29]^.

(3) MSC therapy requires cryopreservation, controlled thawing, and parenteral administration, limiting its use as a routine dermatologic treatment. MSC-sEVs can be incorporated into stable topical formulations (e.g., ointments) suitable for outpatient prescribing and self-application, enabling repeated localized dosing without infusion infrastructure^[30]^.

Collectively, these comparative considerations support MSC-sEVs - especially as a topical formulation - as a more consistent, scalable, and practically deployable alternative to MSC cell therapies for moderate psoriasis.

Although several strategies are being explored to enhance extracellular vesicle (EV) function, including vesicle engineering and surface modification to improve targeting or cargo delivery, this review focuses primarily on native (non-engineered) MSC-sEVs. These vesicles exhibit inherent immunomodulatory activities and currently represent the most extensively characterized EV platform in clinical development for inflammatory skin disorders, including psoriasis. Engineering or functionalization of EVs may alter vesicle composition, physicochemical properties, or biological activity. Such modifications also introduce additional manufacturing, analytical, and comparability requirements. At present, it remains unclear whether engineered EV approaches offer reproducible clinical advantages over native MSC-sEVs. Accordingly, native MSC-sEVs provide a pragmatic and well-defined development pathway for early clinical translation in this therapeutic area^[30]^.

## PRECLINICAL EVIDENCE: TOPICAL MSC-SEVS IN PSORIATIC INFLAMMATION

**Figure 4:**
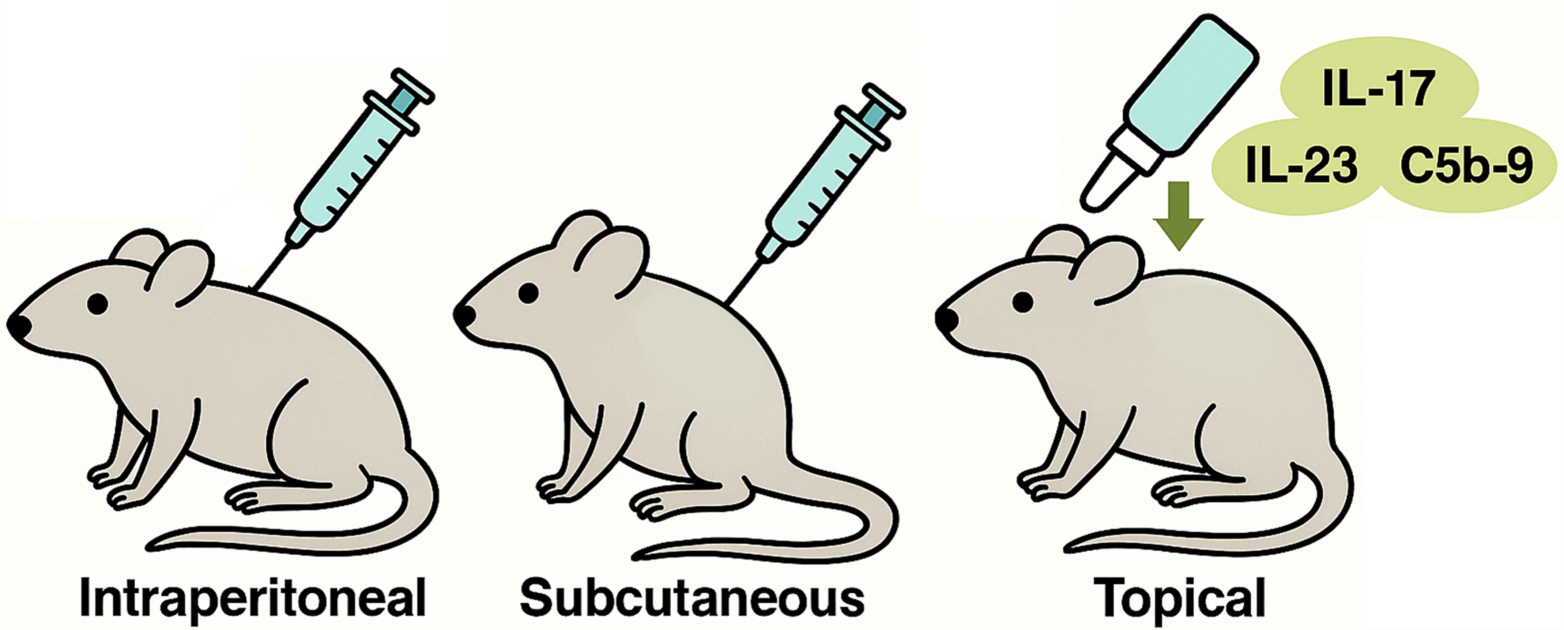
Topical MSC-sEVs Suppress Psoriatic Inflammation. Topical application of MSC-sEVs significantly reduced IL-17, IL-23, and C5b-9 deposition in lesional skin. In contrast, intraperitoneal and subcutaneous injections failed to produce comparable effects, underscoring the unique suitability of topical delivery for localized control of psoriatic inflammation. (Figure was created with AI-assisted technologies). IL-17: Interleukin-17; IL-23: interleukin-23; C5b-9: complement component 5b-9; MSC-sEVs: mesenchymal stem cell-derived small extracellular vesicles.

Preclinical studies using an immortalized clonal MSC source demonstrated that topical application of MSC-sEVs in the imiquimod-induced mouse model of psoriasis significantly reduced IL-17 and IL-23 cytokine expression and terminal complement component 5b-9 (C5b-9) deposition in lesional skin [Figure 4]. Paradoxically, systemic administration in the same model (intraperitoneal or subcutaneous injection) failed to achieve similar effects, underscoring the unique suitability of topical delivery^[31]^.

### Biodistribution of topical MSC-sEVs

**Figure 5:**
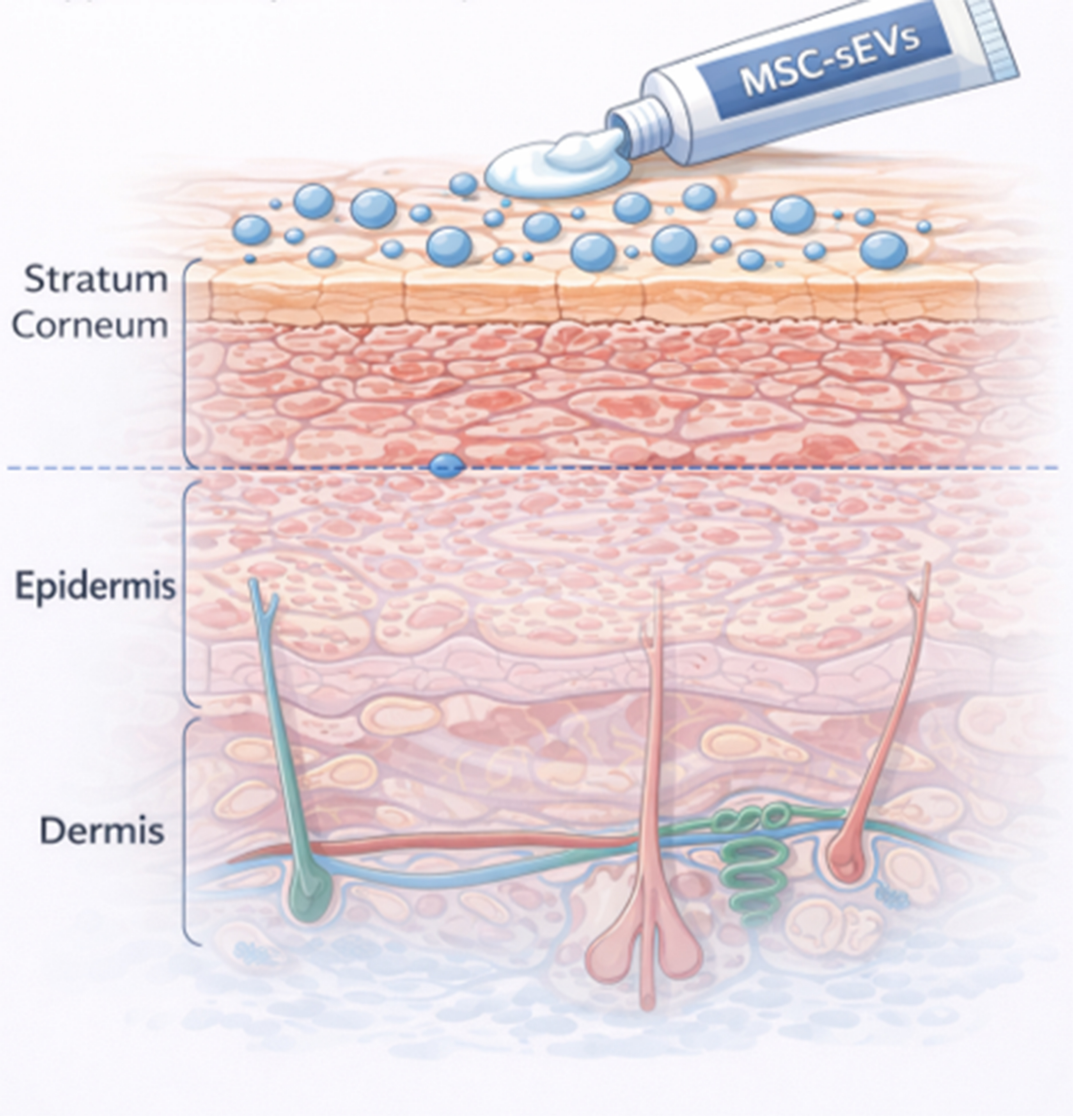
Localization of topical MSC-sEVs to the stratum corneum. Topically applied MSC-sEVs localize exclusively to the stratum corneum, acting at the skin barrier without penetrating deeper epidermal or dermal layers. (Figure was created with AI-assisted technologies). MSC-sEVs: Mesenchymal stem cell-derived small extracellular vesicles.

When MSC-sEVs covalently labeled with a fluorescent dye were applied to human skin explants, fluorescence signals localized exclusively to the stratum corneum and became undetectable within approximately 24 h [Figure 5]. Negligible fluorescence was detected in the underlying culture medium, indicating that topically applied MSC-sEVs remained confined to the outermost epidermal layer and did not penetrate into viable skin compartments^[31]^. The rapid loss of fluorescence is likely due to enzymatic degradation within the stratum corneum, which is rich in proteases^[32,33]^, phospholipases^[34]^, RNases^[35]^, and DNases^[36]^. These enzymes, collectively contribute to the turnover of extracellular material in this barrier layer, suggesting that topically applied MSC-sEVs are short-lived and act locally within the stratum corneum without systemic dissemination.

In contrast, subcutaneously injected MSC-sEVs did not persist in skin tissue and were rapidly degraded^[37]^. Collectively, these findings indicate that topically applied MSC-sEVs remain confined to the stratum corneum, exert localized effects without systemic exposure, and represent an ideal modality for targeting skin-resident inflammatory processes in moderate psoriasis.

### Early psoriatic pathology: complement-driven neutrophil recruitment

A defining early histopathological feature of psoriasis is the Munro microabscess, characterized by neutrophil accumulation within the stratum corneum, the outermost layer of keratinized, non-viable cells^[38-41]^. This lesion represents an early manifestation of innate immune activation at the epidermal barrier preceding visible plaque formation.

Psoriatic plaques show pronounced complement activation, with elevated levels of the chemotactic fragments C3a and C5a and the terminal complement complex C5b-9 detected within the cornified layer^[42-46]^. These complement components, particularly the chemotactic anaphylatoxins C3a and C5a, likely orchestrate neutrophil recruitment into the stratum corneum, establishing the cellular foundation of Munro’s microabscess and initiating an upstream inflammatory cascade that amplifies psoriatic lesion development.

### Barrier-restricted mechanism of topical MSC-sEVs

Since topically applied MSC-sEVs localize exclusively to the stratum corneum**,** with no detectable penetration into viable epidermis or dermis, this indicates that their pharmacological action occurs within this non-viable barrier compartment.

The concurrent presence of activated complement components and neutrophils in the psoriatic stratum corneum suggests that complement activation contributes not only to neutrophil recruitment but also to neutrophil activation and cytokine release. Neutrophils are now recognized as a key source of IL-17, a pivotal cytokine in psoriatic inflammation^[47-49]^.

MSC-sEVs are enriched in CD59, a potent complement regulatory protein that inhibits assembly of C5b-9^[50]^. By delivering CD59 directly to the stratum corneum, topical MSC-sEVs block local complement activation, reducing C5b-9 formation. The mechanistic link between complement inhibition and decreased IL-17 production was clarified in recent studies showing that C5b-9 triggers neutrophil NETosis, which in turn induces IL-17 release^[51]^. This induction was abrogated using a CD59 neutralizing antibody confirming CD59 as the critical quality attribute of MSC-sEV therapeutic effect on psoriatic inflammation.

**Figure 6:**
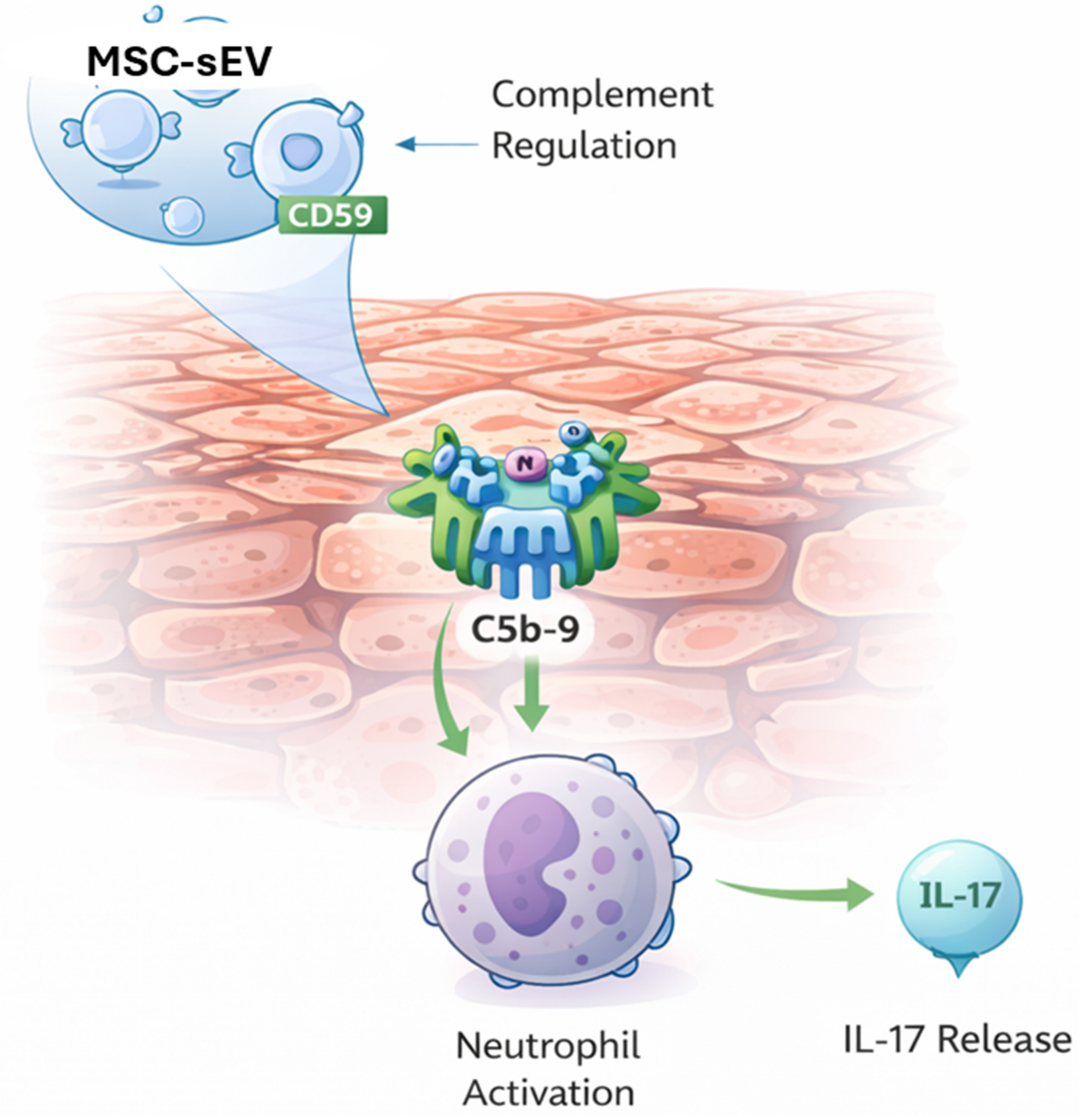
Inhibition of Complement-Induced IL-17 Secretion by MSC-sEV-Associated CD59 in the Psoriatic Stratum Corneum. Schematic representation of the mechanism by which MSC-sEVs mitigate psoriatic inflammation. In the psoriatic stratum corneum, formation of the terminal complement complex (C5b-9) activates neutrophils, inducing NETosis and IL-17 secretion. MSC-sEVs deliver the complement regulatory protein CD59, which inhibits C5b-9 assembly, thereby preventing complement-driven neutrophil activation and IL-17 release - attenuating a key upstream amplifier of psoriatic inflammation at the skin barrier. (Figure was created with AI-assisted technologies). MSC-sEVs: Mesenchymal stem cell-derived small extracellular vesicles; CD59: cluster of differentiation 59; C5b-9: complement component 5b-9; IL-17: interleukin-17.

Collectively, these findings establish that topical MSC-sEVs act within the stratum corneum to inhibit C5b-9-driven NETosis and IL-17 release thereby suppressing a critical early amplifier of psoriatic inflammation at the barrier surface [Figure 6]. Neutralization of CD59 abrogates this effect, confirming that MSC-sEV-associated CD59 mediates the inhibition of complement-induced neutrophil activation^[31]^. This mechanistic localization to the skin barrier provides a strong rationale for topical rather than systemic administration.

Topical delivery offers several mechanistic and practical advantages in this setting:

· High local concentration at the barrier: Topically applied MSC-sEVs are delivered directly to the stratum corneum, where complement activation and NETosis occur, enabling high local exposure at the site of pathology without the dilution inherent to systemic dosing.

· Minimal systemic exposure and off-target effects: Biodistribution studies show that topically applied MSC-sEVs remain confined to and are degraded within the stratum corneum. This barrier-restricted pharmacology contrasts with systemic administration, which exposes multiple organs and carries a greater potential for off-target interactions.

· Practicality for chronic disease: Psoriasis is a chronic, relapsing skin disease typically managed in outpatient settings. A topical MSC-sEV formulation can be self-applied, integrated into existing topical regimens, and does not require infusion facilities, monitoring, or systemic pre-medication, making it more feasible for long-term use in moderate disease.

Finally, we note that psoriasis pathogenesis extends beyond neutrophils. Dysregulation of other immune cell types such as T cells and macrophages, aberrant keratinocyte proliferation and differentiation, enhanced dermal angiogenesis, and intrinsic defects in the epidermal barrier all contribute to plaque formation and chronicity. The direct effects of MSCderived EVs on these additional dimensions of psoriasis remain less well characterized and represent important priorities for future studies.

## PHASE 1 CLINICAL EVIDENCE

The transition of MSC-sEVs into clinical testing was guided by a robust preclinical rationale demonstrating both safety and disease-relevant activity. In an animal model of psoriasis, topically applied MSC-sEVs acted within the stratum corneum**,** where they inhibited complement activation and neutrophil-driven IL-17 release - key early events in psoriatic inflammation. Biodistribution studies using fluorescently labeled MSC-sEVs confirmed that these vesicles remain confined to the stratum corneum**,** with no detectable penetration into the viable epidermis, dermis, or systemic circulation. This localization indicated a mechanism restricted to the non-viable epidermal barrier and suggested a low risk of systemic exposure or toxicity.

To verify clinical safety**,** a Phase 1, single-center, open-label study (ClinicalTrials.gov identifier NCT05523011**)** was conducted to evaluate a topical ointment formulation of MSC-sEVs in healthy volunteers^[30]^. Participants received repeated topical applications over several days, with comprehensive assessment of local and systemic tolerability. The product demonstrated an excellent safety profile: no treatment-related AEs, allergic reactions, or local irritation were observed, and no systemic abnormalities were detected in hematologic or biochemical parameters. Consistent with preclinical data, no systemic absorption of MSC-sEV components was identified.

The favorable safety outcome can be attributed to the intrinsic properties of MSC-sEVs. These vesicles are non-replicative and lack major histocompatibility complex (MHC) class I/II and co-stimulatory molecules such as CD40, CD80, and CD86, minimizing the risk of immune activation or rejection. Moreover, as cell-free biologic, MSC-sEVs are incapable of proliferation or tumorigenic transformation. Together, these attributes confer distinct safety advantages over both live-cell and systemic biologic therapies.

Collectively, the Phase 1 findings establish that topically applied MSC-sEVs are safe, well tolerated, and locally confined, supporting their continued clinical development as a non-immunosuppressive, topical biologic for moderate psoriasis. By combining the biological sophistication of cell-derived immunomodulators with the practicality of a topical formulation, MSC-sEVs represent a new class of skin-targeted therapeutics that could bridge the longstanding treatment gap between conventional topicals and systemic biologics.

## CONCLUSION

**Figure 7:**
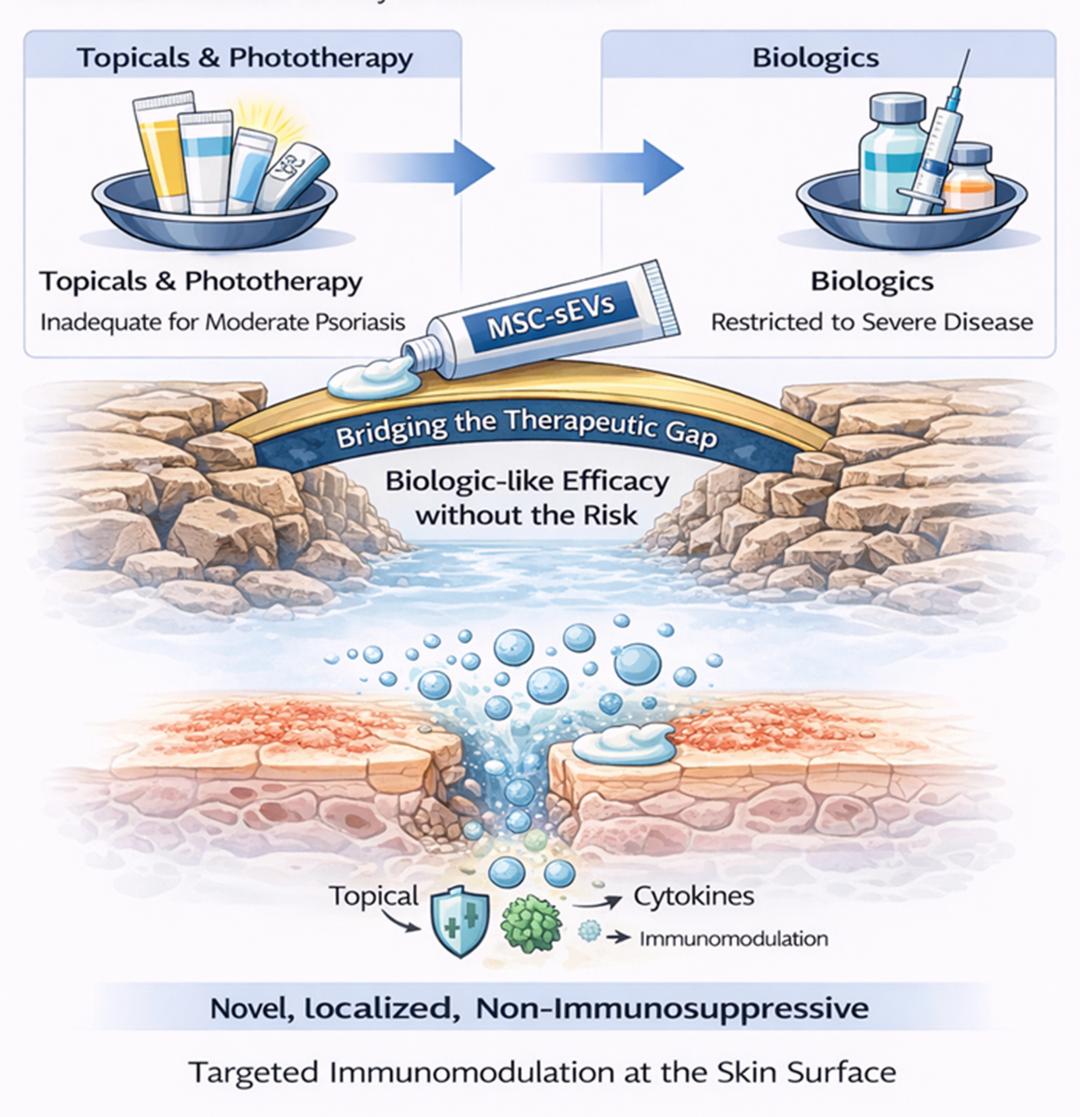
Addressing the Therapeutic Gap in Moderate Psoriasis with MSC-sEVs. Current treatments leave patients with moderate psoriasis undertreated - topicals and phototherapy are inadequate, while biologics are restricted to severe disease. Topically applied MSC-EVs offer a novel, localized, non-immunosuppressive therapeutic option that bridges this gap by delivering targeted immunomodulation directly at the skin surface. (Figure was created with AI-assisted technologies). MSC-sEVs: Mesenchymal stem cell-derived small extracellular vesicles; MSC-EVs: mesenchymal stem cell-derived extracellular vesicles.

MSC-sEVs represent a promising new class of cell-free biologics capable of addressing the unmet therapeutic need in moderate psoriasis [Figure 7]. Patients in this category remain underserved - topical agents and phototherapy provide inadequate disease control, while systemic biologics, although effective, are restricted by cost, safety concerns, and access criteria that reserve their use for severe disease.

Topically delivered MSC-sEVs act locally within the stratum corneum, where psoriatic inflammation originates, exerting potent yet non-immunosuppressive modulation of complement and cytokine pathways. By interrupting early inflammatory events, MSC-sEVs offer a mechanistically distinct approach that reduces inflammation without systemic toxicity.

While large-scale manufacturing remains significant hurdles for clinical translation, emerging strategies offer a clear path toward overcoming these bottlenecks. The use of immortalized MSC lines can effectively mitigate donor-to-donor and batch-to-batch heterogeneity, providing a consistent and scalable source for EV production. Furthermore, the elucidation of specific mechanistic mediators in psoriasis - such as CD59-mediated complement regulation - provides a basis for defining disease-relevant critical quality attributes (CQAs) and robust potency assays. When integrated with Good Manufacturing Practice (GMP)-compliant, closed-system manufacturing processes, these advancements will be essential for ensuring the consistency, safety, and efficacy required for the widespread clinical adoption of MSC-EVs as a novel therapy for psoriasis. Collectively, these features position topical MSC-EVs as an ideal therapeutic bridge for patients with moderate psoriasis: offering biologic-like efficacy with the accessibility, safety, and convenience of a topical treatment.

## DECLARATIONS

### AI and AI-assisted tools statement

During the preparation of this manuscript, the AI tool ChatGPT was used solely for grammar/spelling edits and for rendering assistance in figure preparation. The tool did not influence the study design, data collection, analysis, interpretation, or the scientific content of the work. All authors take full responsibility for the accuracy, integrity, and final content of the manuscript.

### Copyright

© The Author(s) 2026.
